# Oral Bone Tissue Engineering: Advanced Biomaterials for Cell Adhesion, Proliferation and Differentiation

**DOI:** 10.3390/ma12142296

**Published:** 2019-07-18

**Authors:** Alexandra Roi, Lavinia Cosmina Ardelean, Ciprian Ioan Roi, Eugen-Radu Boia, Simina Boia, Laura-Cristina Rusu

**Affiliations:** 1 Department of Oral Pathology, “Victor Babes” University of Medicine and Pharmacy Timisoara, 2 Eftimie Murgu Sq., 300041 Timisoara, Romania; 2 Department of Technology of Materials and Devices in Dental Medicine, “Victor Babes” University of Medicine and Pharmacy Timisoara, 2 Eftimie Murgu sq, 300041 Timisoara, Romania; 3 Department of Anaesthesiology and Oral Surgery, “Victor Babes” University of Medicine and Pharmacy Timisoara, 2 Eftimie Murgu Sq., 300041 Timisoara, Romania; 4 Department of Ear, Nose and Throat, “Victor Babes” University of Medicine and Pharmacy Timisoara, 2 Eftimie Murgu Sq., 300041 Timisoara, Romania; 5 Department of Periodontology, “Victor Babes” University of Medicine and Pharmacy Timisoara, 2 Eftimie Murgu Sq., 300041 Timisoara, Romania

**Keywords:** tissue engineering, oral tissue, biomaterials, scaffolds, biomolecules, cells

## Abstract

The advancements made in biomaterials have an important impact on oral tissue engineering, especially on the bone regeneration process. Currently known as the gold standard in bone regeneration, grafting procedures can sometimes be successfully replaced by a biomaterial scaffold with proper characteristics. Whether natural or synthetic polymers, biomaterials can serve as potential scaffolds with major influences on cell adhesion, proliferation and differentiation. Continuous research has enabled the development of scaffolds that can be specifically designed to replace the targeted tissue through changes in their surface characteristics and the addition of growth factors and biomolecules. The progress in tissue engineering is incontestable and research shows promising contributions to the further development of this field. The present review aims to outline the progress in oral tissue engineering, the advantages of biomaterial scaffolds, their direct implication in the osteogenic process and future research directions.

## 1. Introduction

Both the hard and soft oral tissues play a crucial role in the development and maintenance of the main functions of the human body. Strongly exposed to various external destructive factors (trauma, infections, malignancies), the loss or alteration of these tissues can negatively influence life quality. The maxillofacial and oral cavity areas contain different complex tissues that can successfully benefit from the great potential of biomaterials. The main criteria that can provide an excellent outcome for regenerative medicine are represented by: 1. cells; 2. the existence of appropriate scaffolds and biomaterials that can offer support for different cell types; 3. the addition of growth factors that can contribute to survival and further cell differentiation [1].

In recent years, several biomaterials have successfully contributed to the compensation of functional loss. These biomaterials have similar characteristics to the replaced tissue (corrosion rate, biocompatibility, non-toxicity, specific degradation rate). The development of tissue engineering technologies has achieved its goal regarding the implementation of novel approaches and alternatives of biomaterials in order to replace the oral tissue. This multidisciplinary approach is based on several principles of biology, chemistry, mechanics and materials, in order to obtain the perfect alternative to substitute the missing type of tissue.

The studies conducted in regenerative medicine aim to transform tissue engineering into one of the most important research fields, with successful strategies in order to approach current health issues. Biomaterials have gained a well-deserved place in dentistry and oral surgery, with a wide range of applications in hard and soft tissue regeneration [2], tooth and pulp–dentin regeneration [3,4] and salivary gland lesions [5].

Biomaterials involved in tissue engineering have been improved over the years, and the basic characteristics—such as tissue related toxicity, physical and mechanical strength—have been upgraded. The latest advancements in this field have ensured the achievement of ideal characteristics such as resistance to corrosion [6], non-toxicity and non-carcinogenic properties, bioactivity and appropriate mechanical strength compared to the surrounding tissue type [7]. 

From a health care perspective, the definition of a biomaterial refers to a natural or synthetic material that can be placed into different living tissues without developing an immune reaction [8]. The continuous development of biomaterials needs to accomplish all the interaction mechanisms that occur in different targeted organs, and to mediate cell proliferation and differentiation. Once the biomaterial is placed into a specific tissue, its surface initiates a series of events that determine an immediate interaction with the surrounding cells. This interaction results in the charging of the biomaterial surface energy that will further become an adequate matrix for biomolecule adhesion [9].

With the aid of biomaterials, regenerative medicine has improved the field of tissue engineering. Starting from the use of medical devices or grafts, the progress in this field has led to the development of natural or synthetic biomaterial scaffolds. Biomaterials induce specific regenerative responses of the cells through bioactive molecules, thus successfully replacing the missing tissue.

The advancement in biomaterials reached the third generation and managed to obtain scaffolds that can induce specific cellular responses (adhesion, differentiation, proliferation) [8]. Further research has been focused on the use of biomaterials as scaffolds, combined with growth factors and biomolecules, in order to improve tissue response. Scaffolds serve as an extracellular matrix, a further 3D support for the attachment of stimulated cells. In vitro and in vivo studies have been performed using various biomaterials as scaffolds: natural biodegradable polymers, hydrogels, ceramics, bioactive glass and synthetic polymers [9]. 

Multidisciplinary approaches were needed in order to obtain and stimulate the development of different tissue structures with an appropriate functionality. The key to success is to provide a proper microenvironment that ensures cell adhesion and differentiation. The coating of scaffolds with stem or differentiated cells is a novel and promising method, with an increased degree of complexion, which can also be used in the field of regenerative medicine. Despite the technological solutions provided, scaffolds can sometimes determine immunogenic reactions, degradation and other complications. The initial supposition—according to which, materials are incapable of determining tissue responses and they can only physically substitute the missing tissue—has been proved invalid. The improvement of materials ensured the possibility of physical changes and the existence of certain characteristics such as chemical variability. These aspects prove the biological usage of biomaterials.

In the present review, we aim to outline the advancements made in the field of biomaterials involved in tissue engineering. The characteristics of biomaterial scaffolds provided the possibility of enhancing their chemical and biological properties, thus influencing cell adhesion, proliferation and differentiation. The particularities of the scaffolds such as the surface, porosity and fiber architecture play an important role in further tissue development. Moreover, the dentistry field currently benefits from several alternatives for the oral soft and hard tissue regeneration management, thus successfully improving treatment and surgical outcomes.

## 2. Scaffolds Development: Generalities

Research in tissue engineering focuses on the improvements made in the field of biomaterials which can support the development of further scaffolds used for regenerative purposes. Scaffolds are the key players in tissue engineering through their numerous properties that can induce repair or initiate regenerative processes in different tissue types (Table 1). They can be either natural or synthetic materials that have also been successfully introduced in dental medicine, proving their applicability. The biological properties ensured their usage for multiple purposes such as repairing or stimulating the regenerative process in muscles, bones, nerves, mucosa and skin [10].

A scaffold is described as a three-dimensional biomaterial block, with different designs, which promotes and intermediates multiple functions, such as cell adhesion, due to the characteristics of its surface. Scaffolds must also ensure an adequate environment for growth factors and nutrients in order to stimulate cell differentiation and proliferation. Their degradability rate must provide enough time for tissue regeneration [12].

Scaffolds may be represented by natural or synthetic biomaterials, as long as their main properties influence tissue regeneration by assuring the adequate function of the surrounding cells [13]. The advantage of using biomaterial scaffolds is represented by the fact that a proper tissue response is generated. Biomaterial scaffolds can also successfully substitute autografts or allografts, surpassing their limited indications [13]. Basically, the improvements in this field—especially regarding the development of scaffolds—aim to induce a proper signal in the surrounding tissue cells, through the addition of growth factors, thus generating a cellular response that mediates the differentiation process. 

Scaffolds represent key components for interaction with cells, mainly through their structure, that develops an extra-cellular matrix [14,15,16,17]. Beside the fact that scaffolds are responsible for delivering growth factors and cytokines, their composition and structure play a crucial role in scaffold–cell interactions [17,18,19]. The scaffold–cell interaction is influenced by the pore structure of the scaffold’s surface, one of the characteristics that mediates cell adhesion and differentiation. This characteristic is also implicated in providing the cells with the development of a vascular support [14,16,17]. Studies have shown the involvement of the scaffold’s architecture—including the pore interconnectivity and design—in the success of the regenerative process. Moreover, the cell response is influenced by the chemical characteristics of the surface and by hydrophilicity [20,21,22]. The cell adhesion potential is increased in the presence of a high hydrophilic surface [23,24].

Biomaterials suitable for scaffolds can be divided into different categories based on their nature: natural-based polymers, synthetic-based polymers, ceramics, hydrogels, bioactive glass. The natural polymer’s category includes collagen, chitosan, gelatin and alginate, which are rapidly gaining popularity in the tissue engineering field due to their high biocompatibility [10]. When analyzing the interactions between a natural polymeric scaffold and the targeted tissue, the pore size, porosity, chemical surface changes and fibrous structure have incontestable implications. Synthetic polymers are represented by polylactic acid, polyglycolic acid and polylactic-co-glycolide [10]. These types of synthetic polymers possess different biomechanical properties, chemical surfaces and degradation rates associated with various cellular responses. 

Scaffold biomaterials must have a specific configuration, 90% porous, with strict pore diameters and connections, which allow cells to adhere and proliferate, generating new tissue and developing a proper vascular system [25]. Although researchers aimed to create the ideal biomaterial that could fulfill all the criteria, biocompatibility and non-toxicity remain the primary desirable properties of a biomaterial scaffold [26]. The importance of generating a non-inflammatory reaction remains the first step for the initiation of the regenerative process. The absorption rate of the biomaterial scaffold is an important aspect that must be taken into consideration, as it must permit a sufficient period of time for the tissue to regenerate and completely regain its normal functions [27]. The studies conducted until now have proved the fact that the elasticity of the scaffold increases with its volume, but, the increase in volume produces reduced permeability, with important consequences on the porosity of the biomaterial scaffold. This suggests that, in order to obtain a high resistance and elasticity, cell adhesion will be influenced by the porosity alterations [28]. All the advantages and disadvantages of various biomaterials must be considered in order to identify the most suitable one for a certain type of tissue. 

## 3. The Development of Angiogenesis

Regenerative medicine is focused on the successful integration of biomaterial scaffolds. This success is guaranteed by a proper vascularization development in specific areas, ensuring the bidirectional transport of growth factors, nutrients and the formation of novel tissue cells [29]. Cell adhesion and survival are the key players in obtaining novel tissue. The prior implantation of various cells and growth factors onto the scaffold surface, before its placement, creates a favorable environment for further cell proliferation and differentiation. 

Angiogenesis is defined by the formation of novel blood vessels from pre-existing ones and it involves numerous mechanisms that are coordinated by endothelial growth factors, signaling molecules and specific extracellular matrix proteins [30]. Several studies focused on this issue mention the addition of growth factors and signaling molecules on biomaterial scaffolds [30,31,32]. However, the growing problem regarding their exact time of action and efficiency still remains, as the rapid degradability and uncontrolled release represent two of their disadvantages.

The advancements in biomaterials ensured the development of different materials with angiogenic actions stimulated by their own degradation [33,34,35,36,37]. Several studies have been performed in this field and angiogenic actions have been reported when using bioactive glass as a scaffold [38]. These aspects have been reviewed by Gorustovich et al. [39]. Bioactive glass has been shown to possess angiogenic effects in both bone and soft tissue engineering [29,40,41,42]. 

The addition of growth factors (such as vascular endothelial growth factor and fibroblast growth factor) is essential for the formation of neovascularization due to the involvement of these molecules in developing a novel vascular system along with the already existing vessels [43]. The challenge consists in the fact that, although these vessels are the key to success in regenerative medicine, their growth requires permanent monitoring in order to avoid the appearance of vascular malformations [44,45].

Multiple studies were performed in order to identify the proper coating of biomaterial scaffolds—which has adequate angiogenic effects—by promoting the vascular endothelial growth factor. The study conducted by Day et al. [46] was focused on quantifying the effect of bioglass on the fibroblast population and concluded that in the case of bioglass-coated scaffolds, fibroblasts were stimulated to secrete an increased amount of vascular endothelial growth factor. This fact resulted in an increased vascularization development, compared with the control group without bioglass-coated scaffolds.

## 4. Oral Bone Tissue Engineering

Bone tissue is described as a complex structure, mostly consisting of hydroxyapatite and collagen [47]. Although it has an increased regenerative potential, in some cases, such as large defects, surgery is necessary in order to correct and properly stimulate the regenerative process. The conventional approach to solving these cases involves the use of bone grafts (autografts, allografts or xenografts). The tissue engineering industry and the improvements in the biomaterials field have overcome a series of disadvantages by introducing the use of polymers scaffolds (natural and synthetic), ceramics, bioglass, cell coating and the addition of growth factors. Bone tissue engineering aimed to fulfill the required characteristics of the bone augmentation process, by using different matrices and combining biomaterials in order to obtain a favorable environment for the adhesion, proliferation and differentiation of osteoblasts. The key to success in bone tissue engineering consists in the scaffold’s properties, its customization, biomechanical properties and added signaling factors that will influence osteoinduction and osteogenesis [48].

The term ‘’scaffold’’ refers to the development of a biomaterial with the main purpose of acting as a support surface, thus creating a favorable environment that can restore the physiological and histological characteristics of the injured tissue [49,50,51]. 

Three-dimensional scaffold printing allows for the addition of growth factors that can be distributed at different levels. A study performed by Philippi et al. [52] showed that the cells applied onto the bone morphogenic protein underwent osteogenic and differentiation processes, while the cells that were placed in the outer part showed no differentiation process. The facts that growth factors have a shortened in vivo lifespan and that the biomechanical properties of the scaffold must be well documented, are two aspects that must be taken into consideration. Other types of cells that showed great potential in the regenerative process are the mesenchymal stromal cells, the adipose tissue-derived stem cells and the oral cavity mesenchymal stromal cells. The integration of these types of cells on biomaterial scaffolds in in vitro studies showed an increased potential in the biofabrication of bone tissue [53].

A variety of biomaterials have been introduced in bone tissue engineering, thus developing a wide category of scaffolds. Ceramics (calcium phosphate and tricalcium phosphate) are very popular due to their similar properties to the bone tissue. Bioactive glass, zirconium oxide and silicon dioxide are other examples of ceramics used in bone tissue engineering. Out of the synthetic polymers, polylactic acid, polylactic-co-glycolic acid and polycaprolactone have attracted researchers’ interest. The natural category of polymers: chitosan, collagen, alginate, gelatin or glycosaminoglycans, represents a popular option in regenerative medicine. 

Oral bone tissue engineering must overcome certain challenges related to the oral region, in order to obtain the perfect regenerative biomaterial. An important issue that should be taken into consideration is the fact that the biomaterial is exposed to the oral environment, which includes the presence of various pathogens. Due to this aspect, the fabrication of the biomaterial scaffold should consider several issues regarding its antimicrobial properties, the release of bioactive factors and degradation ratio [54]. 

The improvements in science and biomaterials have introduced a novel category of materials, known as smart materials, with a variety of applications [55]. These materials are characterized by reproducible and stable variations of at least one characteristic when in contact with exogenous stimuli (shape memory materials, conductive polymers, temperature-responsive polymers) [56]. 

The target of bone tissue engineering and regenerative medicine based on biomaterials is to develop proper scaffolds that can fulfill all the characteristics required by certain tissues in order to promote cell adhesion, proliferation, differentiation and angiogenesis. Biomaterials for bone tissue engineering must stimulate and properly replace the physiochemical characteristics of the surrounding tissue through a special design that creates an adequate environment.

## 5. Advancements in Biomaterial Scaffolds

### 5.1. Natural Polymers

Chitosan represents a deacetylated form of chitin that originates from the exoskeleton of crustaceans. It is a copolymer made of D-glucosamine and N-acetyl-D-glucosamine bonds and *β* bonds [57]. Certain enzymes such as lysozyme, lipases and glycosaminidases are responsible for the depolymerization of chitosan [58]. From a structural point of view, chitosan resembles glycosaminoglycans and has an important role in cell-to-cell adhesion through collagen fiber interactions. During its depolymerization process, chitosan has major antimicrobial actions, and it is described as having excellent biocompatibility with all types of tissues. A study performed by Moorthi et al. [59] concluded that although chitosan has an important osteoconductive property, it displays a low osteoinductive action. It manages to control the proliferation of osteoblasts and mesenchymal cells and it is implicated in the initiation of the neovascularization process. All chitosan’s properties are due to its chemical groups (amino and hydroxyl). Biodegradability, absorption and solubility rates are characteristics given by the amino groups, thus transforming chitosan into an appealing option for tissue engineering [60,61]. An advantage of this natural polymer is its high molecular weight, making it a strong viscosity agent and allowing it to act as a pseudoplastic biomaterial in an acid medium [62]. The molecular weight and the degree of deacetylation are two main properties that influence the biodegradation characteristics of this material. Both in vitro and in vivo experiments have shown that, in case of a higher molecular weight, the biodegradation process is slower [63].

In tissue engineering, the use of chitosan permits the development of specific scaffolds that can offer support and successfully induce the regenerative process. Also, one of the most important characteristics is the scaffold’s porosity. A high porosity scaffold allows proper cell adhesion and proliferation and ensures an adequate surface for the cells to adhere and create connections through the surrounding existing pores. The correct pore distribution facilitates proliferation and the pore connections provide an environment for cell growth and for products’ interchange [11]. The mechanical properties of the chitosan scaffolds represent a disadvantage, mainly because the membranes are brittle and rigid, with low resistance. In order to overcome these shortcomings, crosslinking agents are used in order to build an improved scaffold with more efficient mechanical properties.

The use of chitosan in tissue engineering and repairing is due to the fact that it is easily processed in multiple forms such as fibers, sponges, hydrogels and films. The ability to provide different shapes, in order to be placed in various locations, makes this type of biomaterial very popular. Its chemical structure is similar to some polysaccharides and has the possibility to undergo changes in order to suit different host tissue types [11].

Based on its biological and physiochemical properties, the use of chitosan scaffolds in bone tissue regeneration is appealing. Its matrix offers an increased biocompatibility and also creates a favorable environment for cell interactions, making it one of the first-choice materials [64,65]. Chitosan matrices can undergo changes when using different osteoinductive materials, such as calcium phosphate, calcium sulfate [66,67] and hydroxyapatite [68]. The goal is to obtain an osteogenic effect based on the addition of organic and inorganic materials [69].

Studies were performed regarding the evaluation of chitosan composites in osteoblast cultures, and an in vitro analysis on the interaction of the MC3TC cell line with chitosan and tripolyphosphate membrane showed the same results as in the controls. Chitosan composites with calcium phosphate showed a significant release of morphogenic protein type-2, proving an increased compatibility of these biomaterials with osteoblasts [66,70] (Table 2). On experimental models, chitosan scaffolds were analyzed for their potential in the bone regeneration segment and the use of chitosan hydrogel proved to have an effective regenerative potential [71]. Other similar results were reported in cases where chitosan and hydroxyapatite were used to fill a bone defect, showing a larger number of osteogenic markers in the experimental group [72]. Chitosan and nanohydroxyapatite composites gained interest in tissue engineering due to their ability to determine an osteogenic response in osteoblasts. Some studies also reported an increased bone regeneration rate in rabbits that were evaluated by tomography after 8 weeks [73].

Collagen scaffolds are another type of natural polymer with an important action on osteoblasts, creating a favorable environment for their adhesion. Once the colonization of osteoblasts occurs, the degradation of the collagen scaffold is initiated, and the cells will entirely replace the material (Table 2). A study performed by Wang et al. [74] evaluated the regeneration of an extraction socket in dogs. The study was based on the use of two different composites and the results showed that in the group in which collagen scaffolds were used, the process of osteogenesis was better than in the other group, but the mechanical properties were worse [74]. Ber et al. [75] based their study on different changes in collagen following the addition of other materials. Their conclusion was that collagen scaffolds underwent changes following dehydrothermal treatment and that carbodiimide crosslinking showed a more effective potential for cell adhesion and proliferation. A lower cell proliferation was found in collagen scaffolds treated with glutaraldehyde. An increased proliferation, as well as the initiation of the mineralization process, was found in the group represented by calcium phosphate-treated collagen [75].

Alginate is a natural polysaccharide, widely used as a biomaterial, with a great potential to be used as a scaffold. Moreover, it has numerous properties—such as biocompatibility, non-toxicity, biodegradability—which are responsible for its absent immune response [47]. Over time, alginate found its use in the food industry, biomaterials for orthopedic purposes [76] and in the field of tissue engineering. Alginate scaffolds originate from a cross-linking process that mostly uses calcium-based substances which transform alginate into a hydrogel. Its success in tissue engineering is a consequence of the addition of various cells and growth factors on scaffolds, thus facilitating the regeneration rate. Alginate scaffolds, wound dressing materials that contain bioactive molecules [77,78,79] and transplantation of stem cells have improved the tissue engineering industry [80]. Currently, it has proved its contribution in angiogenesis and in the delivery of growth factors or other substances [81,82]. This biomaterial has shown its efficiency through its properties, out of which the molecular weight, concentration and purity play a key role in regenerative medicine by influencing cell adhesion, proliferation and differentiation. Studies have shown that alginate scaffolds with high molecular weight and improved mechanical properties act as proper scaffolds for hard tissue engineering [47] (Table 2). The influence on the cell population is influenced by adding different substances to alginate hydrogel scaffolds. Rubert et al. [83] conducted a comparative study in which the action of alginate hydrogel scaffolds and hyaluronic acid on the bone tissue was evaluated. Although alginate and hyaluronic acid have similar characteristics, the results showed that alginate hydrogels had a greater influence in inducing cell adhesion and proliferation, determining an increase in the level of osteocalcin and alkaline phosphatase in comparison to the hyaluronic acid group [83]. It appears that the significant impact on the osteogenic process is justified by the carboxylic acid contained in the alginate scaffolds.

### 5.2. Synthetic Polymers

Synthetic polymers have several advantages compared to the natural ones. Some of these advantages are represented by the controlled degradation rate and by the size and weight that can be designed to properly fit into the targeted tissue. As these types of scaffolds can be changed for a particular design, their mechanical strength and degradation rate can be adjusted in order to obtain maximum performance. Synthetic polymers were designed for the development of proper scaffolds for bone tissue engineering. They are saturated poly-a-hydroxy esters such as polylactic acid (PLA), polyglycolic acid (PGA) and polylactic-co-glycolide (PLGA). The advantage of using these polymers lies in the fact that they are characterized by different properties and their mechanical strength and biodegradation are sometimes higher than those of natural ones [10]. There are noticeable differences between the three categories of synthetic polymers—PGA being a more hydrophilic polymer with an increased degradation rate, while PLA has more hydrophobic characteristics. Polyglycolic acid has three forms: PLLA, PDLA and PDLLA. The copolymerization of these polymers results in more efficient biochemical characteristics, and an increased mechanical strength and degradation rate [84]. Polylactic-co-glycolide is a synthetic polymer that has proved its efficiency in bone regeneration studies. The success of the polymeric scaffolds consists in their structural architecture, porosity, and the chemical characteristics of the surface. All of these aspects have a major impact on the cellular response (Table 2). In a study performed by Woo et al. [85], PLLA scaffolds were modified by increasing their nanofibers, with important effects on the osteoblastic differentiation and biomineralization rate. Another study performed by Badami et al. [86] evaluated the fibrous PLA scaffold surface in comparison to the smooth one and concluded that the fibrous PLA scaffold surface has a greater potential for cell adhesion, with influences on osteogenic differentiation. The importance of the pore size was outlined by several studies that have shown an increased degree of adhesion and proliferation of osteoblasts on scaffolds with pore sizes ranging between 400 and 600 μm and with a high porosity [87,88]. In this case, osteoblasts’ proliferation occurred on both surfaces of the scaffold, as well as in the inner part–due to its large pores—while in the cases of PLGA scaffolds with smaller pore sizes, osteoblasts adhered only onto the surface [88]. One of the advantages of synthetic polymers is represented by the fact that their surface can be changed in order to create a favorable environment. Several studies concluded that modifying the surface of PLGA scaffolds and etching them with NaOH, improves their surface characteristics and roughness [89]. Other means to improve the surface are through changing the PCL scaffold surface by etching it with a solvent based on acetone 90%, resulting in an enhancement of the surface characteristics along with an increased osteogenic differentiation [90]. In other studies, the scaffold’s surface was subjected to O_2_ plasma treatment and the results showed an important cell adhesion, proliferation and differentiation process, in cases of PCL and PLLA scaffolds that underwent this type of surface treatment [91,92]. Another approach in improving the cell adhesion rate through surface alteration is by coating the PLLA scaffolds’ surfaces using laminin, gelatin and cholesterol. In all of these cases, the results showed that cell adhesion and proliferation was higher [93,94].

In synthetic polymer scaffolds, the pore size and porosity are important parameters related to tissue engineering. Structure is also one of the main elements that influences the adhesion, proliferation and differentiation of specific cells. The interconnectivity of the pores is also an important aspect that permits cell nutrition, vascularization and oxygen diffusion [95]. The scaffold’s surface modification has a proven direct influence on cell adhesion, migration, proliferation and differentiation.

Most of the scaffolds contain a single pore size or porosity type, but native biological tissues are characterized by a layered pore size architecture. A major goal of tissue engineering scaffolds is to resemble the native tissues, by using the biomimicry concept. Solutions to best match the natural tissue pattern have been offered by high-resolution 3D printing of different polymers. A novel concept in the fabrication of porous polycaprolactone-based functionally grated scaffolds—using EHD-jet 3D printing technology—might represent an option [109].

### 5.3. Stem Cells Carriers

The combination of various biomaterials with stem cells represents the future in regenerative medicine. Combining the structural and mechanical properties, biocompatibility and degradation degree with stem cells offers the possibility to achieve a proper environment for the osteoinductive process. Researchers report a positive outcome for in vitro testing, but further research needs to be performed in order to describe all the molecular actions which occur. The goal is represented by the association of different biomaterial scaffolds with stem cells in order to obtain certain cell responses. The use of collagen scaffolds and stem cells has been studied in vitro, in vivo on animals and also in human clinical trials. Kawase et al. showed that this type of scaffold promoted cell adhesion and proliferation while being used for periodontal treatment [110]. In other studies, the combination of synthetic scaffolds, PDLLA and collagen respectively, has important effects on the differentiation of mesenchymal stem cells. Similar results have been reported for the PDLLA and gelatin combination scaffold [111]. 

According to Engler et al., microenvironments play an important role in stem cell lineage specification. Naive mesenchymal stem cells are shown to specify lineage and commit to phenotypes sensitive to tissue elasticity. Soft matrices that mimic the brain are neurogenic, stiffer matrices that mimic muscle are myogenic, and rigid matrices that mimic collagenous bone prove osteogenic. Reprogramming of these lineages is possible during the initial week in culture, but afterwards the cells commit to the lineage specified by matrix elasticity. These facts have significant implications in understanding the physical effects of the in vivo microenvironment and also for therapeutic uses of stem cells [112].

Bone marrow stromal cells have proven suitable for repairing defects and damages. On the other hand, many biometric materials are used to improve and correct the body defects. Nanofibers are widely used in tissue engineering, as scaffolds in wound healing and wound dressing. Chitosan/polyethylene oxide nanofibers can be a suitable replacement for routine wound coverages. A study conducted by Rahimi et al. presents a combination of these methods and concludes that combining two treatment methods leads to better results, when tissue engineering and cell therapy are involved [113].

Further research is required in this field in order to fully understand and associate cellular responses to this category of scaffolds.

### 5.4. Novel Scaffold Design and Concepts

Due to the fact that the main property which qualifies a scaffold for bone tissue engineering is its biocompatibility, natural polymer-based scaffolds consisting in proteins, polysaccharides, minerals, growth factors, etc., and the interaction between scaffolds and cells, have to be considered in the first place. Encouraging future perspectives of natural polymer-based scaffolds for bone tissue engineering must be mentioned [114].

The field of bone tissue engineering is constantly developing—more and more variants of scaffold design and conception are becoming available. Among the newest ones, the biomimetic porous scaffolds based on triply periodic minimal surfaces (TPMS)—which involve optimization to match the elastic properties of human’s bone—have been described by Vijayavenkataraman et al. [115].

Several design methods for TPMS scaffolds have been mentioned in the literature, which are able to satisfy multiple requirements including porosity, Young’s modulus, and pore size. Three different applications of TPMS: tissue specific scaffolds, scaffolds for stem cell differentiation and functionally graded scaffolds with biomimetic functional gradients are assessed by Vijayavenkataraman et al. in their study [115].

With the advent of 3D printing, a new era has begun. Many scaffolds are now fabricated using 3D printing methods and especially 3D bioprinting, which is a promising and useful technology.

One of the biggest challenges for 3D bioprinting is dentistry. Because of the complexity and the multicellular interaction, the challenges in this area are great. Recently, progress has been made in 3D printing of biocompatible materials, seed cells, and supporting components into complex 3D functional living tissue, but, for now, 3D bioprinting remains limited to the regeneration of dental pulp and the tooth germ [116].

Drug functionalized scaffolds are intended for improving the local delivery of osteoprotective drugs, in order to reduce the loading dose and the unwanted systemic complications. Poly-(ε) caprolactone (PCL)-laponite-strontium ranelate composite scaffold was studied by Prabha et al. It has been proven to support growth and osteogenic differentiation of human marrow-derived stromal stem cells. The in vitro and in vivo experiments showed its possible applications in bone regeneration in the fields of orthopedics and dentistry [117].

The influence of static or dynamic magnetic fields on biological systems suggested new opportunities in tissue engineering, namely magnetic scaffolds. D’Amora et al. analyzed the effect of the application mode of a time-dependent magnetic field on the behavior of human mesenchymal stem cells seeded on 3D additive-manufactured poly(3-caprolactone)/iron-doped hydroxyapatite nanocomposite scaffolds. It has been proven that extremely low frequency improves the proliferation, synthesis and secretion of growth factors, stimulating angiogenesis and promoting bone formation [118]. The above-mentioned research may be considered as a preliminary approach to analyzing the effects of the application of an external time-dependent magnetic field in conjunction with 3D nanocomposite magnetic scaffolds, and could open new perspectives for the application of magnetic fields and cell-laden scaffolds for bone tissue engineering. 

### 5.5. Our “Future Perspectives”

**Figure 1:**
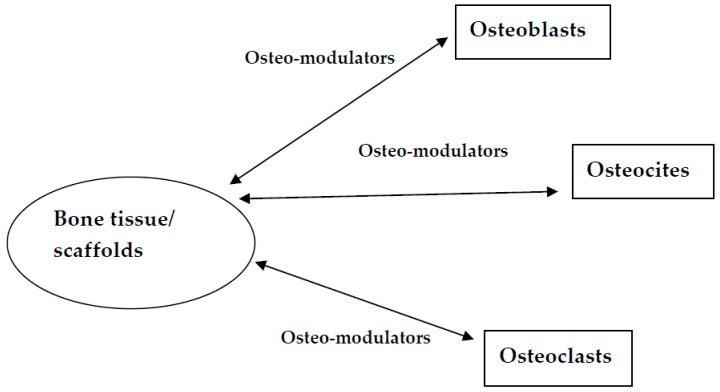
Schematic representation of the mechanisms involved.

Obviously, scaffolds belong to the future of the osteoinductive mechanism. The perfect scaffold does not yet exist, but a quality one involves passing the clinical situation and regenerative response through the receptors and activating physiological mechanisms similar to those produced by osteoclasts, osteocites and osteoblasts (Figure 1). Scaffolds that mimic the structure and composition of bone tissue and cells play an important part in bone tissue engineering applications.

## 6. Conclusions

Regenerative medicine and tissue engineering have come a long way, and their advantages ensure the opportunity to stimulate healing or the replacement of tissues with a biocompatible alternative. The development and advancements in the biomaterials field have achieved successful outcomes in relation to cell adhesion, proliferation and differentiation. Because of the maxillofacial region’s complexity, which implies important challenges for tissue engineering, the biomaterials used need to fulfill certain biological characteristics, mechanical properties and osteinductive processes. The complete elucidation of the interactions between cells and biomaterial scaffolds leads to promising outcomes in bone regeneration. Whether natural or synthetic polymers, the main properties regarding biocompatibility, non-toxicity and chemical surface characteristics need to promote and enhance a proper integration process. The advantages and disadvantages of various scaffolds require evaluation in order to obtain efficient results. Using and combining biomaterial scaffolds with growth factors and biomolecules represents the key element for cell stimulation and development of vascularization. Natural polymers can serve as scaffolds due to their biocompatibility and osteinductive action, but their usage depends on their mechanical properties. Synthetic polymers have a well-controlled surface, composition and physiochemical characteristics that transform them into appealing choices in bone tissue engineering. Several up-to-date choices are available, such as 3D-printed functionally grated or magnetic scaffolds. Currently, modern medicine lacks the optimal scaffold to suit every clinical situation. In various clinical cases, scaffold selection must be based on the documented interactions between various biomaterials and targeted cell types. Despite the numerous challenges, the use of biomaterial scaffolds in bone engineering offers a wide perspective and opportunity to overcome the current grafting gold standard. The objective is to develop compatible choices for a vascularized, mechano-chemical and functionally appropriate tissue replacement.

## Figures and Tables

**Table 1:** Main properties of biomaterial scaffolds used in regenerative medicine [11].

Properties	Importance
Biocompatibility	The scaffolds should not determine rejection responses from the body
Non-toxic/Non-carcinogenic	Their components or degradation products should not cause biological responses
Chemical stability	Chemical alterations should not occur, at least during the regenerative process
Mechanical properties	Mechanical properties must complete tissue requirements; resistance and weight should also be similar
Adequate chemical surface	The surface characteristics should favor cell adhesion, differentiation and proliferation
Shape, dimension and design	They should fit in the targeted tissue, stimulating the regenerative process
Absorbability and degradability	Absorbable, with an adequate degradability rate in concordance with the tissue regenerative/repair process

**Table 2:** Of scaffolds used in bone tissue engineering.

Type of Scaffold	Properties	References
Chitosan + Alginate	Increased apatite deposition, efficient protein absorption	[96]
Chitosan + Carboxymethyl cellulose	Stimulates biomineralization	[97]
Chitosan + gelatin	Increases biomineralization and decreases the degradation rate	[98]
Chitosan + alginate	Stimulates differentiation and mineralization	[99]
Chitosan + collagen	Increases the vascularization rate	[100]
Collagen	Increased biocompatibility, non-toxic, easy to manipulate and deliver growth factors	[101,102]
Polylactic acid	Absorbable synthetic polymer, variable degradation rate, low mechanical strength	[103,104]
Polyglycolic acid	Absorbable synthetic polymer, rapid degradation, low mechanical strength	[103,104]
Polylactic-polyglycolic acid	Control surface, pore size and morphology of the scaffold, growth factor delivery, hydrophobic	[84,104]
Polylactic-polyglycolic acid	Improved cell adhesion, proliferation and differentiation	[105]
PLLA	Increased cell adhesion and proliferation	[106]
PLLA	Increases osteoblast differentiation, influences biomineralization	[107]
PCL	Promotes osteogenic differentiation, cell proliferation and infiltration	[108]
